# Utilization of telehealth services in low- and middle-income countries amid the COVID-19 pandemic: a narrative summary

**DOI:** 10.1080/16549716.2023.2179163

**Published:** 2023-02-20

**Authors:** Biplav Babu Tiwari, Aneesh Kulkarni, Hui Zhang, Mahmud M. Khan, Donglan Stacy Zhang

**Affiliations:** a Department of Health Policy and Management, College of Public Health, The University of Georgia, Athens, GA, USA; b Woodward Academy, Atlanta, GA, USA; c School of Public Health, Sun Yat-sen University, Guangzhou, China; d Division of Health Services Research, New York University Long Island School of Medicine, Mineola, NY, USA

**Keywords:** Telehealth services, low- and middle-income countries, COVID-19, effectiveness, cost

## Abstract

**Background:**

During the current period of the pandemic, telehealth has been a boon to the healthcare system by providing quality healthcare services at a safe social distance. However, there has been slow progress in telehealth services in low- and middle-income countries with little to no evidence of the cost and effectiveness of such programmes.

**Objective:**

To provide an overview of the expansion of telehealth in low- and middle-income countries amid the COVID-19 pandemic and identify the challenges, benefits, and costs associated with implementing telehealth services in these countries.

**Methods:**

We performed a literature review using the search term: ‘*country name* AND ((telemedicine[Title][Abstract]) OR (telehealth[Title][Abstract] OR eHealth[Title][Abstract] OR mHealth[Title][Abstract]))’. Initially, we started with 467 articles, which were reduced to 140 after filtering out duplicates and including only primary research studies. Next, these articles were screened based on established inclusion criteria and 44 articles were finalised to be used in the review.

**Results:**

We found telehealth-specific software being used as the most common tool to provide such services. Nine articles reported patient satisfaction of greater than 90% with telehealth services. Moreover, the articles identified the ability to make a correct diagnosis to resolve the condition, efficient mobilisation of healthcare resources, increased accessibility for patients, increased service utilisation, and increased satisfaction as benefits of telehealth services, whereas inaccessibility, low technological literacy, and lack of support, poor security standards and technological concerns, loss of interest by the patients, and income impacts on physicians as challenges. The review could not find articles that explored the financial information on telehealth programme implementation.

**Conclusion:**

Although telehealth services are growing in popularity, the research gap on the efficacy of telehealth is high in low- and middle-income countries. To better guide the future development of telehealth services, rigorous economic evaluation of telehealth is needed.

## Introduction

Coronavirus disease 2019 (COVID-19) has emerged as the most consequential global health problem since the 1918 influenza pandemic, resulting in more than 6 million deaths worldwide as of March 2022 [1]. Since being declared a global pandemic, COVID-19 has caused significant impacts on healthcare and socialcare systems and society as a whole. A systematic review by Moynihan and colleagues in 2021 found that healthcare utilisation decreased by about a third during the pandemic, with considerable variation, and greater reductions among people with less severe illnesses [2]. Moreover, a survey by the World Health Organisation found a higher rate of disruptions of health services in low- and middle-income countries as compared to that in high-income counties [3].

During the current period of the pandemic, the use of telehealth has rapidly expanded in many countries, especially in developed countries [4,5]. The term telehealth covers a wide spectrum of services provided at a distance without direct physical contact with the patient, either using provider-to-patient or provider-to-provider communications [6]. Although the concept of modern telehealth dates back to the 1960s with the projects launched by the National Aeronautics and Space Administration (NASA) and the Nebraska Psychology Institute, in recent years, with increased Internet access and digital devices, it has become ubiquitous in parallel with a growing need for more convenient, accessible, and cost-effective health care [4,7]. Moreover, the COVID-19 pandemic set the world for the telehealth revolution that we are now experiencing. Telehealth programmes limited the physical contact between the patients and service providers for inpatient and outpatient services, thus reducing the community and nosocomial spread of the coronavirus [6,8]. Furthermore, it allowed providers with mild COVID-19 symptoms and/or who were exposed to the coronavirus to remain at home and take care of their patients remotely [4,9].

The global growth in telehealth has been steady and much slower in low- and middle-income countries due to the lack of sustainable models, limited financial resources, health policy priority of the country, poor knowledge, and limited access to telehealth technologies [4,10–12]. Much of the evidence on the utilisation of telehealth in low- and middle-income countries has been focused on identifying general barriers to telehealth use rather than on evaluating the actual telehealth programs implemented in those regions. In times of COVID-19 where the already poor healthcare system of low- and middle-income countries are on the verge of collapse, the evidence on telehealth programmes can help implement a programme to uplift their healthcare systems. Thus, this narrative summary aims to provide an overview of the expansion of telehealth in low- and middle-income countries since the onset of the COVID-19 pandemic and identify the challenges, benefits, and costs associated with implementing telehealth services in these countries.

## Methods

**Figure 1.**
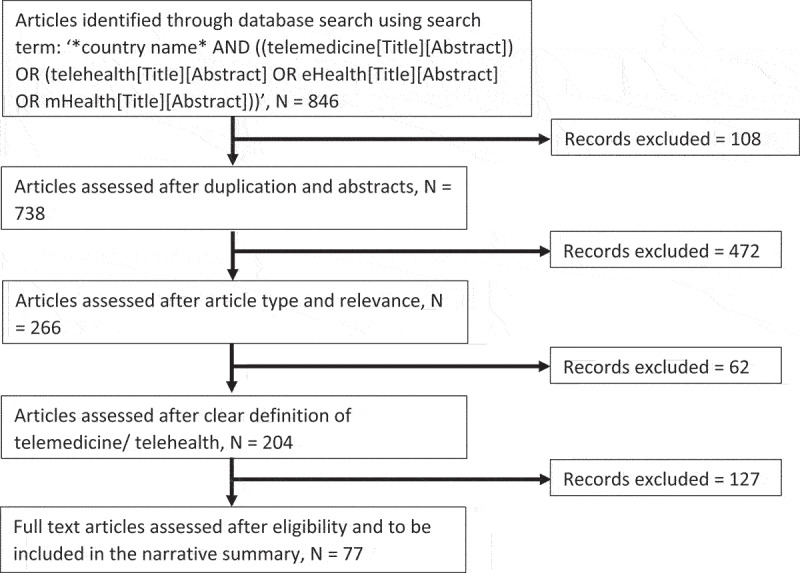
Article selection process for the narrative review.

A literature review was performed for the articles related to telehealth and telemedicine on PubMed using the search term: ‘*country name* AND ((telemedicine[Title][Abstract]) OR (telehealth[Title][Abstract] OR eHealth[Title][Abstract] OR mHealth[Title][Abstract]))’. Two researchers – BT and AK – independently conducted the systematic search and article retrieval. Searches using the references cited in the retrieved articles were also performed. We used the following keywords: ‘Telehealth’, ‘Telemedicine’, ‘eHealth’, and ‘mHealth’, and a list of countries. Health Resources and Services Administration defines telehealth as ‘the use of telecommunications and information technologies to share information and provide clinical care, education, public health, and administrative services at a distance’ [13]. The list of low- and middle-income countries was referenced from World Bank [14]. A total of 846 articles were identified through database searching, and after removing the duplicated articles, we obtained a total of 738 articles. Next, we filtered for article type i.e. primary research studies relevant to the target country resulting in a total of 266 articles. Furthermore, these 266 articles were screened if they had mentioned telemedicine/telehealth as their keywords resulting in a total of 204 articles. Finally, all 204 articles were reviewed by the two researchers and the final number of articles for the review was 77. The final selection used articles based on specific intervention area areas, tools used for telemedicine/telehealth, target country, and relevance to the study. Inclusion criteria included 1) articles written in English, 2) articles published between January 2020 and December 2022, and 3) having a clear definition of telehealth/telemedicine, specific intervention area, and tools used for telemedicine/telehealth. We excluded duplicated articles, abstracts, and those with contents not related to the primary focus of the study. Figure 1 shows the details regarding the article search, and inclusion/exclusion criteria used for screening and selection of relevant studies.

As mentioned earlier, 77 articles were reviewed for final inclusion in the study. The findings from each article were arranged in the different columns according to their theme (constructed as result in the upcoming section) using Excel. It helped to arrange the data in a structured format and develop a theory based on the information identified.

## Results

### Telehealth practice in low- and middle-income countries

In this section, we discuss the general telehealth practices in low- and middle-income countries during the time of the COVID-19 pandemic.

Out of the 77 articles reviewed, 1 was from a low-income country, Niger, and 25 from lower-middle-income countries: Bangladesh (2), Bolivia (1), Egypt (5), India (13), Iran (2), Tunisia (1) and Zimbabwe (1), and 51 from upper-middle-income countries: Argentina (3), Brazil (11), China (16), Columbia (1), Georgia (1), Lebanon (2), Libya (1), Mexico (6), Montenegro (1), Paraguay (1), Serbia (1), Thailand (2) and Turkey (1). Higher utilisation of telehealth services was observed in countries with higher socioeconomic levels.

Out of the 77 studies, 11 were randomised controlled trials, 50 were cross-sectional studies, 10 were longitudinal studies, 1 was a qualitative study from India, and 5 were case studies. Among the 11 randomised controlled trials, two were conducted in a lower-middle-income county, Egypt, and the remaining 9 studies were in upper-middle-income countries. Similarly, the study from Niger, a low-income country, was a cross-sectional study with a sample size of 335. There were 14 cross-sectional studies from lower-middle-income countries and the remaining 36 cross-sectional studies were from upper-middle-income countries. Out of the 10 longitudinal studies, lower-middle-income and upper-middle-income had 5 studies each. Finally, two case studies were conducted in India, one in Egypt, whereas the remaining 2 case studies were from Georgia and Brazil.

Looking at the tools used for providing telehealth services, applications/software were most prevalent in these studies; 21 out of 77 articles reported the use of some form of software and applications. Out of these 21 studies, 16 used telehealth-specific software, new or existing, such as VOIP telephony, TRS, Tiantanzhixin app, DBS tell programming, Te Acompañamos, Sofia’s mobile app, Line Platform, TeleDiab and Coronaid and the remaining 5 studies do not specify the software and/or application names. Moreover, 14 out of the 16 telehealth-specific software were being used in the upper-middle-income compared to only 2 in the lower-middle-income countries and none in the low-income countries. Audio-visual tools were the second most prevalent telehealth approach used. Out of the 11 studies that reported audio-visual methods, 8 mentioned an audio-based approach only for telehealth consultation whereas 3 combined the audio and video consultation. In addition, these audio-visual tools were more prevalent in lower-middle-income countries where 5 audio-based approaches and 1 audio-visual mixed approach were from the region. Similarly, chat-based software such as WhatsApp (16), Zoom (11), WeChat (6), SMS (4), Facebook Messenger (2), google hangouts (2), and Skype (4) were used to deliver services.

The published articles indicate that 66 were newly adopted telehealth initiatives during the COVID-19 pandemic, while the remaining were either expansions of previous telehealth services or evolution of the existing system with some added features during the pandemic situation.

### Interventions fields of telehealth programs

Table 1 provides a summary of the intervention fields that the telehealth services applied in the 77 reviewed articles. The literature used telehealth services mostly to understand the palliative care for chronic diseases such as cancer followed by management of COVID-19.

**Table 1.** Intervention fields assessed by the reviewed literature.

S.N.	Intervention Fields	Frequency (N = 77)
1	Palliative care for chronic diseases	20
2	COVID-19	12
3	Pediatrics	9
4	Mental health and wellbeing	6
5	Cardiovascular diseases	5
6	Neurology	4
7	Orthopedics	3
8	Maternal and Child Health	3
9	Epilepsy	3
10	Others	6

### Benefits of telehealth services

Different benefits of telehealth services identified in the selected literature were discussed as follows.

#### Higher level of patient satisfaction with telehealth service utilisation

Out of the 77 articles reviewed, 22 had reported levels of patient satisfaction with telehealth. In half of these studies, more than 90% of the participants reported being satisfied with telehealth services whereas, in the remaining studies, the percentages of participants satisfied with the services ranged from 66.24% to 87%. The main reasons for dissatisfaction identified were unavailability of multidisciplinary advice over a single call, poor internet connections, difficulty in telehealth tool use (such as software, and mobile applications), no detailed history taken, poor consultation, and no provision to select a doctor of patient’s choice, lack of video option, limited time for consultation, and medicine prescription not being sent in addition to the verbal advice.

Moreover, nine out of 77 explored the provider’s perceptions of telehealth practices. The majority of these articles reported that physicians had good knowledge, a positive attitude, and high satisfaction. Two of these studies reported that 90% of the healthcare providers are satisfied with the telehealth services and are open to including such services in their practice during the pandemic. One article reported that though the physician had skepticism initially, they showed a positive inclination towards it over time. However, physicians reported difficulty in performing a remote clinical evaluation, and poor quality of images, sound, and video transmission.

#### Confident diagnosis to resolve health conditions

One of the major benefits of telehealth services identified was the correct diagnosis to reach the required health outcome through its utilisation. Telehealth was applied in different settings and was found to be as effective as in-person consultation visits to diagnose and prescribe appropriate treatments. A study by Moyo and Madziyire 2020, in Zimbabwe, found that healthcare providers were able to make accurate diagnoses, and conditions were resolved in 49.3% and 52.2% respectively using telehealth services [15]. Another study found that 90% of physicians were receiving images of their patients’ wounds and radiological data and among them, 90% were satisfied with the quality of sound and video [16]. The physicians in the same study expressed interest in continuing such a telehealth approach as a substitute for in-person visits to receive images and videos of the wounds and radiological data. Moreover, a study in Bolivia used a telehealth approach to estimate epilepsy prevalence during the COVID-19 pandemic and report it as a valuable tool in performing epidemiological research [17]. Several studies found telehealth to be effective in medical conditions related to back pain, cervical symptoms, shoulder range of motion (ROM) measurement, respiratory illness, ketonemia/ketonuria, autism care, rehabilitation services, epilepsy, Parkinson’s disease, diabetes, hypertension, and intensive care unit (ICU) for making proper diagnosis and provision of appropriate treatment for them [18–28].

#### Efficient healthcare service resource utilsiation

In addition to correct diagnosis, telehealth in low- and middle-income countries was found to be effective in terms of efficient mobilisation of limited healthcare resources. Even in high-income countries, during the COVID-19 pandemic, the management of already scarce healthcare resources was a challenge. The pandemic largely reduced the availability of adequate healthcare personnel to handle the COVID-19 patient burden as well as disrupted the supply chain of the required medical equipment and items [29,30]. Telehealth services helped hospitals and other service sites to resolve the management of healthcare personnel deficits to some extent by allowing healthcare providers and patients to have consultations during their feasible times and locations [9,31]. The virtual consultation reduced the chances of the nosocomial spread of COVID-19 among patients and reduced the likelihood of getting infected among already scared healthcare personnel [6,8,25,32]. For example, a study in Tunisia found telehealth services to be time-saving as well as to avoid school absenteeism [33]. Telehealth approach was prominent in cardiac emergencies, orthopedic outpatient departments (OPDs), and services for pregnant women [22,34,35]. Moreover, it acted as a gatekeeper by screening the patient before they visit a health practitioner or a facility for specialty care [15].

#### Increased healthcare accessibility for patients

Telehealth has also been proven to be effective not only for the providers but also for the patients to access healthcare services in low- and middle-income countries. Generally, people in low- and middle-income countries face the barrier of long travel distances, high travel expenses, and high costs to visit specialty care. But these issues were eliminated or reduced immensely through telehealth services [16,22]. Studies in low- and middle-income countries found telehealth services reduced travel time, prevented unnecessary visits and travel expenses associated with it, detected complications early, provided an early response to emergencies, e-prescriptions, more personalised attention, and increased accessibility in rural areas [16,21,25,32,36–41]. For example, a study in Brazil reports average travel time saved as a result of telehealth was 289.6 minutes, which accounted for R$106.67 saved [41].

#### Increased service utilisation by patients

The benefit of correct diagnosis by the physicians with minimum hospital resource utilisation and increased accessibility by the patient increased healthcare service utilisation. Especially in times of COVID, telehealth led to increased access and utilisation of healthcare services as remote access does not require practice from a physical space, the ability to provide services with flexible hours, remote consultation, and patient-centered services [42]. The user-friendly nature of telehealth services in terms of interface design and navigation system increased the easiness to connect and consult with healthcare providers, ultimately leading to the adoption of such services and increased healthcare utilisation [43]. Studies have shown varying degrees of increased utilisation with as high as 802% for COVID-19 teleconsultations in Brazil, a 40-fold increase for services related to neurosurgery in Montenegro, and a fivefold increase for COVID-19 management in China [44–46].

### Challenges of telehealth services

In this section, we discuss different challenges of telehealth services identified from the reviewed literature.

#### Inaccessibility to telehealth services

One of the main challenges for telehealth services in low- and middle-income countries was the inaccessibility to telehealth itself. Low- and middle-income countries face the challenges of access to communication devices such as smartphones, computers, or any other electronic communication devices, access to internet services, and web-based applications [15,16,19,24,39]. These components are key building blocks of a basic telehealth program and without their proper access, one cannot operate efficient and equitable telehealth services that cover the entire population of the country. A study in Egypt assessed the challenges of telehealth services, where 37 patients reported a lack of smartphones and 29 patients reported a lack of poor internet connectivity out of 200 patients [47]. Another study in Mexico found the most frequent issues to be interruptions (14.7%), problems with Internet connectivity (12.8%), and poor-quality video/audio (12.2%) [48].

#### Lack of technological literacy and support

Technological illiteracy and lack of support are the next major challenges in operating telehealth services in low- and middle-income countries. Due to these issues, patients were not holding the phone at the proper angles for improving the quality of video and photos. In addition, the issues such as a lack of understanding of terminologies, unclear and incomprehensible buttons, understanding advice, difficulty in navigating around the apps, and lack of support structures for telehealth service use, made digital diagnosis much more difficult. These issues could lead to potential misdiagnosis. A survey in Lebanon assessed the barriers to telemedicine use, where 91.7% of physicians reported the inability to perform an adequate physical examination as one of the barriers [49]. Other technical issues such as wrong number registration, blocking of commercial messages, and no internet communication had further adverse impacts on telehealth service operations in low- and middle-income countries.

#### Poor security standards of telehealth programs

Any telehealth services are at risk of a security breach from hackers. Low- and middle-income countries with existing operating issues around telehealth services and poor health systems, poor security standards, underdeveloped policies, insufficient authenticity and reliability, and lack of advanced health technologies are factors leading to a high risk of a security breach. These factors make the information systems weak and easy for hackers to breach and get access to patients’ records and release them. A study in Egypt reported that out of 200 participants, 5 patients expressed the possibility of privacy breaches in the future as barriers to telemedicine use, and another study in Lebanon reported that 3 patients out of 242, expressed their distrust of the technology [38,47].

#### Loss of interest in telehealth service utilisation among patients

All the challenges of telehealth mentioned above in combination with factors such as lack of access to patients in rural areas, inappropriate timing of phone calls, hassle during re-appointment, difficulty in explaining advice to patients as well as difficulty in receiving and interpreting the health advice by patients lower the interest of patients in remote consultations. Lack of spontaneous, in-the-moment interaction with a provider, makes patients and caregivers lose their interest in telehealth services. Moreover, few studies found no significant differences in healthcare outcomes after the telehealth service implementation [50–52]. For example, a study in Brazil found no statistical difference in the level of HbA1c post telehealth program implementation to reduce the triglyceride level for type 2 diabetes [50]. Furthermore, a study in Tunisia found a lower willingness of parents to accept telemedicine as a model of care [33].

#### Income impact on physicians

Physicians also lose interest in providing telehealth services as it creates an extra burden for them to ensure the delivery of quality services. In many cases, they would need to explain and reexplain the health conditions and advice multiple times, for which physicians are not compensated. A study in Lebanon found that about 93% of participating physicians saw the adverse impact of the COVID-19 pandemic on their income, whereas only 16.9% of those who used telemedicine revealed getting paid an additional amount to their regular salaries for telemedicine consultations [53].

### Cost

One study each in Bangladesh and Brazil provided little information on the cost of telehealth services. We found that the average cost to assess the telehealth services in Bangladesh is 532 Tak and 62% of the participants were satisfied to pay the fee to gain the telehealth services [54]. Similarly, in Brazil, the mean labor costs per medical and nursing teleconsultation were Int$ 24 and Int$ 10, and on average it costs Int$ 14 to telemonitor a patient with a daily call for 7 days [55].

## Discussion

In general, the reviewed literature suggests that the provision and use of telehealth services are more prevalent in higher-income countries than low-income countries with 51 studies from upper-middle-income countries. We found telehealth-specific software and/or applications to be the most employed communication tools to provide telehealth services. In more than one-third of these telehealth services, a specific application was developed to operate the telehealth services which allowed healthcare providers to connect with their patients via audio calls, video calls, or even text messages. The remaining ones used publicly available chat-based software such as WhatsApp, and Facebook Messenger as well as call and text services of the phone network to connect with their patients and provide telehealth services.

Only one-fourth of the reviewed literature explored the patients’ satisfaction, where, half of them reported the percentage of patients satisfied to be greater than 90%. Moreover, even a lower number of studies reported providers’ perception i.e. one-tenth, and found physicians to have good knowledge, positive attitude, and high satisfaction with telemedicine practices with few studies reporting as high as 95% satisfaction and acceptance of telemedicine practices during the pandemic. Similarly, the reviewed literature identified the benefits of telehealth services as the ability to make a confident diagnosis to resolve the condition, efficient mobilisation of healthcare resources, increased accessibility for patients, increased service utilisation, and increased satisfaction. Despite the benefits of telehealth services, the literature found challenges of telehealth services such as inaccessibility, technological literacy and lack of support, poor security standards and technological concerns, loss of interest by the patients, and income impacts on physicians.

In the reviewed literature, though we found telehealth services to be effective in many low- and middle-income countries, few studies found it to have no significant difference when compared with the existing system. Studies in Bangladesh on behavioral intention, in Brazil on mitigating the psychological impact of COVID-19, cerebral palsy, and Parkinson’s disease, in China on dementia, hypertension, and type 2 diabetes management, on emergency mHealth use, on retinopathy of prematurity and psychological distress, in Montenegro on neurosurgical care, in Paraguay on postoperative follow-up, in Thailand on type 2 diabetes and Turkey on autoimmune hepatitis, phenylketonuria, breathing exercise, type 2 diabetes, and mental health found a significant improvement in the healthcare services when telehealth services were employed compared to no implementation during the COVID-19 pandemic [23,24,27,41,43,44,56–68]. However, studies in Egypt on anticoagulants and myocardial infarction,in Georgia on self-injury, in Tunisia on children and adolescent arthritis, in Argentina on epilepsy, and in Brazil on type 2 diabetes management found no significant differences in healthcare service utilisation and overall health outcomes with the implementation of telehealth services [33,36,47,50,51,69]. Moreover, additional issues such as lack of detailed history taking, inability to motivate patients properly and lack of adherence to medications, low GDP of the country, high referral rates, and limited experience with communication methods further reduce the efficacy of telehealth services in such economies [22,45,48,70,71].

One of the major gaps identified from the reviewed literature was the lack of cost information for the implemented telehealth programs. We found ample information on the positive impact and challenges of the programs being implemented and we can use them to develop an efficient telehealth program that provides maximum benefits for as minimum risk as possible. However, if we do not have information on the cost, we can only assess the effectiveness of such programs. Despite their effectiveness, if the cost is too high to bear and proves to be less cost-effective than the existing program, then we cannot implement the telehealth programs. A few studies assessed the cost of implementing telehealth programs in low- and middle-income countries. A study conducted in Bangladesh and India in 2017 found telephone-based support for the management of pressure ulcers among people with spinal cord injuries to be more cost-effective than the usual hospital-based care [72]. However, during the time of the COVID-19 pandemic when telehealth services proliferated and were used for different interventions, we have very limited lack evidence on the cost information of implementing such telemedicine programs.

The limited nature of the systematic reviews and studies on the efficacy of telehealth services impacted the result of this narrative summary. Thus, caution needs to be taken when interpreting the findings due to the limitations of the literature. Similarly, the studies were conducted in various settings and countries with different healthcare systems. Thus, their findings cannot be generalised to other settings. Moreover, only 11 out of 77 studies used any form of experimental study design to assess the effectiveness of telehealth services, impacting the quality of the reviewed studies. Despite the limitation, our review had few strengths as well. Our focus on middle- and low-income countries highlights the research gap and need to initiate and improve telehealth services in those regions. Moreover, it can be used to guide future research and develop programs to close the research gaps.

## Conclusion

Although telehealth services are growing in popularity, especially during the COVID-19 pandemic, the evidence for their efficacy is still limited in low- and middle-income countries. However, despite the limited literature and challenges such as inaccessibility, technological literacy and lack of support, poor security standards and technological concerns, loss of interest by the patients, and income impacts on physicians, associated with it, telehealth services in low- and middle-income countries are found to be effective. It has helped those countries by increasing physicians’ ability to make a confident diagnosis to resolve the condition, efficient mobilisation of healthcare resources, increased accessibility for patients, increased service utilisation, and increased satisfaction. However, there is a need for the economic evaluation of telehealth services in the pandemic scenario to better guide future directions in low- and middle-income countries.

## Paper context

Telehealth services rose to popularity during the COVID-19 pandemic. However, we found that although telehealth services are growing in low- and middle-income countries with studies exploring the benefits and challenges to its implementation, we cannot find articles that explored the financial aspect to back up its effectiveness. Thus, there is a need for the economic evaluation of telehealth services in the pandemic scenario to better guide future directions in telehealth development in low- and middle-income countries.
